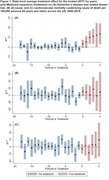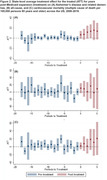# State‐level effect of Medicaid expansion on Alzheimer’s disease and related dementias mortality

**DOI:** 10.1002/alz.087740

**Published:** 2025-01-09

**Authors:** Jeffrey Wing, Jenna Rajczyk, Julie Strominger, Parvati Singh

**Affiliations:** ^1^ Ohio State University, Columbus, OH USA

## Abstract

**Background:**

With the rapid aging of the US population, the prevalence of dementia is projected to double. The enactment of the Affordable Care Act and Medicaid expansion could create opportunities for detection and classification of dementia. There are trends of increasing dementia mortality, however, it is unknown whether Medicaid expansion increased the reporting of dementia as the underlying cause of death (UCOD) or as a multiple cause of death (MCOD) among the elderly.

**Methods:**

State‐level Alzheimer’s disease and related dementias (ADRD) mortality data for those 65 years and older were downloaded from CDC WONDER for 2000‐2019. ADRD was classified as ICD‐10 codes: F01, F03, and G30. Staggered difference‐in‐difference analysis was done to estimate the ADRD mortality rate pre‐ and post‐Medicaid expansion. An overall average treatment effect for the treated (ATT) was estimated along with estimation for ATT by post‐period. Mortality measured as UCOD and MCOD were evaluated separately. Results were compared to all‐cause and cardiovascular disease (CVD) mortality for the same period.

**Results:**

A total of 29 out of 50 states expanded Medicaid by 2019. Post expansion, ADRD mortality increased by 9.02 per 100,000 persons (95% CI: 1.81, 16.23). The change in mortality (UCOD) was most pronounced by two years post expansion, gradually increasing each year (two‐years post ATT: 10.02; 95% CI: 3.51, 16.52; five‐years post ATT: 13.58; 95% CI: 2.82, 24.34; Figure 1a). MCOD ADRD mortality demonstrated a similar pattern as UCOD ADRD mortality (ATT: 11.31; 95% CI ‐1.42, 24.05; Figure 2a). This trend was not observed across the same period for UCOD CVD mortality (ATT: 0.80; 95% CI: ‐12.42, 14.02) and the post‐expansion difference observed for UCOD all‐cause mortality lacked precision in comparison to dementia mortality (ATT: 7.43; 95% CI: ‐23.12, 37.98).

**Conclusions:**

ADRD as the UCOD increased following state‐level Medicaid expansion, but this increase was not observed similarly for CVD mortality nor all‐cause mortality. Additionally, the same effect was not observed for ADRD as a MCOD. The lag between expansion and the increase in mortality may arise from increased detection through Medicaid supported care and in‐turn being listed or correctly identified as the underlying cause of death.